# Wenshenyang recipe treats infertility through hormonal regulation and inflammatory responses revealed by transcriptome analysis and network pharmacology

**DOI:** 10.3389/fphar.2022.917544

**Published:** 2022-08-08

**Authors:** Lan Xie, Shuai Zhao, Xiaoling Zhang, Wenting Huang, Liansheng Qiao, Delin Zhan, Chengmei Ma, Wei Gong, Honglei Dang, Hua Lu

**Affiliations:** ^1^ Medical Systems Biology Research Center, Tsinghua University School of Medicine, Beijing, China; ^2^ National Engineering Research Center for Beijing Biochip Technology, Beijing, China; ^3^ Chengdu University of Traditional Chinese Medicine, Chengdu, China

**Keywords:** wenshenyang recipe, infertility, network pharmacology, transcriptome analysis, GO enrichment analysis, pathway enrichment analysis, molecular docking

## Abstract

The Wenshenyang recipe (WSYR) has the effect of treating infertility, but the mechanisms underlying this activity have not been fully elucidated. In this study, network pharmacology and RNA sequencing were combined, with database-based “dry” experiments and transcriptome analysis-based “wet” experiments used conjointly to analyse the mechanism of WSYR in the treatment of infertility. In the dry analysis, 43 active compounds in WSYR and 44 therapeutic targets were obtained through a database search, 15 infertility pathways were significantly enriched, and key targets, such as ESR1, TP53, AKT1, IL-6, and IL-10 were identified. Then the wet experiments were performed to detect the expression changes of the 412 genes from 15 infertility pathways identified by dry analysis. HK-2 cells were treated with the three herbs of WSYR and subjected to targeted RNA sequencing. Based on the results, 92 of the 412 genes in 15 infertility pathways were identified as DEGs. Additionally, key targets, such as ESR2, STAT1, STAT3, and IL6, were also identified in the wet experiments. RT-qPCR experiments further verified that WSYR played an anti-inflammatory role by upregulating *IL-4* and *IL-10* and *Epimedium brevicornu* Maxim (Yinyanghuo) showed broader effect than *Drynaria fortunei* (Kunze) J. Sm (Gusuibu) and *Cistanche deserticola* Y.C.Ma (Roucongrong). By screening compounds of WSYR using molecular docking models of ESR1 and ESR2, it was further found that xanthogalenol in Gusuibu, arachidonate in Roucongrong, and anhydroicaritin in Yinyanghuo had good affinity for estrogen receptors. These findings provide evidence for an estrogen-regulating role of the three herbs in WSYR.

## Introduction

According to WHO analyses, 186 million individuals (including 48 million couples) worldwide suffer from infertility (Rutstein and Shah, 2004; Boivin et al., 2007; Mascarenhas et al., 2012). Studies have shown annual increases in the incidence of infertility in recent years (Petraglia et al., 2013), and it has become an important factor affecting human health and family stability. Factors affecting both male and female fertility include hyperprolactinism, hypogonadism, cystic fibrosis, systemic diseases, infections and lifestyle-related factors (Vander Borght and Wyns, 2018). The causes of female infertility mainly include ovulation disorders, blocked fallopian tubes, and cervical factors (Vander Borght and Wyns, 2018). The most common cause of female infertility is ovulation failure, which occurs in 30%–40% of infertile women (Nahid and Sirous, 2012). Specifically, it includes anovulation caused by pituitary secretion disorders and ovulation disorders caused by endocrine disorders. Causes of male infertility include abnormal sperm, blocked sperm delivery, and immune factors (Vander Borght and Wyns, 2018). Sperm abnormalities lead to impaired spermatogenesis, impaired maturation, blocked sperm transport ducts, and abnormal gonads, which are major causes of male infertility (Wang et al., 2020). Additional causes of male infertility mainly include abnormal semen, blocked sperm delivery and immune factors.

Assisted reproductive technology (ART) has been used in the treatment of infertility. More than 5 million children worldwide have been born through *in vitro* fertilization and other ART interventions (Messerlian and Gaskins, 2017). However, ART is technologically sophisticated and expensive, such that it is unaffordable or even unavailable in many countries and regions, especially low- and middle-income areas.

As an important category of complementary and alternative medicine, traditional Chinese medicine (TCM) has been widely used in treating infertility in recent decades. Compared with ART, TCM has the advantages of greater accessibility, lower cost, fewer adverse reactions, and higher safety (Huang and Chen, 2008). A study reported that the Wenshenyang recipe can promote the proliferation and differentiation of shoot stem cells and promote the synthesis of cartilage and cartilage matrix during the early limb development of the embryo, which is closely related to the formation and development of the foetus (Xue et al., 2021). The Wenshenyang Recipe consists of three Chinese medicines: *Cistanche deserticola* Y.C.Ma (Roucongrong), *Epimedium brevicornu* Maxim (Yinyanghuo) and *Drynaria fortunei* (Kunze) J. Sm (Gusuibu). All three herbs have similar effects, *i.e.*, warming the kidney and strengthening yang.

Modern pharmacological studies have proven that Yinyanghuo has a wide range of hormone-like effects, regulates the function of the hypothalamus-pituitary-gonad axis, and has the effect of “stimulating yang” in animal models of yang deficiency (An et al., 2015). Yinyanghuo can improve sperm motility in rats and protect against the epididymal damage caused by chemotherapy (Cao et al., 2008). Yinyanghuo also plays a role in reducing the levels of proinflammatory mediators and cytokines (Saba et al., 2020). Roucongrong and its active ingredient echinacoside (ECH) can enhance the biosynthesis of testosterone by upregulating the expression of a variety of steroid-generating enzymes, improving poor sperm quality and reducing testicular toxicity in rats (Jiang et al., 2016). ECH can block hypothalamic androgen receptor (AR) activity and increase the secretion of luteinizing hormone and testosterone, thereby increasing sperm number, to treat oligospermia (Jiang et al., 2018). Gusuibu strengthens muscles and bones and has immunomodulatory activity (Jeong et al., 2005). However, due to the complex components of herbs and the complicated process of infertility development, the mechanism of WSYR for treating infertility is still unclear.

Network pharmacology is a discipline that predicts the active ingredients in TCM prescriptions and explains the potential mechanism of action of TCM prescriptions from a systematic perspective. The concept of holism and focus on systems conforms to the characteristics of TCM and is a more suitable method for studying the multicomponent, multitarget, and multipath mechanisms of traditional Chinese medicine (Li and Zhang, 2013). However, the results predicted by network pharmacology are only speculative results based on available data, not validated effects. Therefore, we aimed to combine network pharmacology with gene expression profiling of TCM-treated cells. Transcriptome analysis was performed by RASL-seq, which combines the RNA annealing, selection, ligation (RASL) strategy and next-generation sequencing (Li et al., 2012). This technology can simultaneously detect the expression of thousands of genes after drug treatment (Shao et al., 2019) and effectively verify the large-scale gene data set predicted by network pharmacology.

In this study, the potential anti-infertility targets of WSYR were obtained through network pharmacology analysis. Pathway enrichment analysis was performed to identify the key pathways of WSYR in the treatment of infertility, and a “TCMs-components-targets-pathways” network map was constructed. Afterwards, transcriptome analysis was utilized to examine the expression of genes in the predicted pathways after drug intervention. Altogether, the key targets of WSYR in the treatment of infertility were identified, and its mechanism of action was explored.

## Materials and methods

### Collection of active ingredients of TCMs and their potential targets

The TCM System Pharmacology Database (TCMSP, https://tcmspw.com/tcmsp. php) (Ru et al., 2014) was used to collect the chemical components of WSYR with the keywords “roucongrong”, “yinyanghuo” and “gusuibu” respectively. Compounds with oral bioavailability (OB) ≥ 30% and drug-like properties (DL) ≥ 0.18 were selected as active components.

The potential targets of each compound were obtained through the TCMSP database. The Entrez ID, and gene symbol of each target were collected.

### Gene ontology analysis and pathway enrichment analysis

ClueGO (Bindea et al., 2009) was used to analyse the GO biological processes and enriched Reactome pathways of different gene sets. The bubble diagram and bar chart were drawn by the Bioinformatics Online Visualization Tool (http://www.bioinformatics.com.cn).

### Cell culture

HK-2 cells were obtained from the National Experimental Cell Resource Sharing Platform (Wuhan, China). SK-OV-3 cells were obtained from Procell Life Science and Technology Co.,Ltd (Wuhan, China). All cells were maintained in DMEM (Gibco, Grand Island, NY) containing 10% foetal bovine serum (Gemini, Woodland, CA) and 100 U/mL penicillin–streptomycin (Gibco, Grand Island, NY) at 37°C.

### Preparation of medicinal extracts

Roucongrong (origin: Neimenggu), Yinyanghuo (origin: Guangdong) and Gusuibu (origin: Jilin) were purchased from Anguo Changda Chinese Herbal Medicine Co., Ltd. All three TCMs were powdered and extracted by a Soxhlet extractor (Extraction Unit B-811, Buchi, Switzerland) with 90% ethanol. Then, the solvent was concentrated in an electrically heated blast drying oven (GZX-9070MBE, Boxun, China) at 45°C. Subsequently, the concentrate was lyophilized with a freeze dryer (ALPHA1-2Dplus, Christ, Germany), weighed and stored at -80°C for later use.

### Transcriptome sequencing

Transcriptome sequencing were performed as previously described (Shao et al., 2019). Briefly, HK-2 cells were cultured in a 384-well plate for 24 h and treated with the three herbal extracts for 24 h. Then, the cells were lysed, incubated at room temperature for 10 min, and stored at -80°C. A total of 412 genes were detected by RNA annealing, selection and ligation with a high-throughput pipetting platform. The ligated products were amplified by PCR and sequenced by a gene sequencer (HiSeq X Ten, Illumina, United States). The data discussed in this publication have been deposited in NCBI’s Gene Expression Omnibus and are accessible through GEO Series accession number GSE202626 (https://www.ncbi.nlm.nih.gov/geo/query/acc.cgi?acc=GSE202626).

### Sequencing data processing

First, the high-throughput sequencing platform data files were converted into raw data for base identification analysis. Subsequently, the original data were filtered by more than 3 base sequence mismatches. Finally, the DESeq software package was used to identify differentially expressed genes (DEGs) with fold change (FC) > 1.5 or <0.67 and *p* value < 0.05. The R package ggplot2 was used to construct a volcano plot of the DEGs (Anders and Huber, 2010).

### Construction of a protein-protein interaction network and screening of key targets

All DEGs were analysed by the STRING database to obtain protein–protein interactions. Protein–protein interaction networks were constructed by Cytoscape (Shannon et al., 2003). The cytoHubba plug-in included in Cytoscape was used for target topology analysis to obtain the main topological parameters of the PPI network. The sum of degree, betweenness and closeness after the standardization of deviation was used as the indicator to determine the key targets.

### RT-qPCR analysis

Total RNA was extracted with all in-one mini spin columns (Epoch Life Science, Fort Bend, TX). Reverse transcription into cDNA was performed using a high Capacity RNA-to-cdna kit (Invitrogen) and qPCR was carried out using the SYBR FAST qPCR Kit (Kapa Biosystems, Wilmington, MA). Finally, the 2^−ΔΔCT^ method (Nolan et al., 2006) was used to calculate the data. The primer sequences are provided in Supplementary Table S1.

### Molecular docking of estrogen receptor α (ESR1) and estrogen receptor β (ESR2)

The protein crystal structure of ESR1 and ESR2 were downloaded from the Protein Data Bank (PDB, https://www.rcsb.org/) for molecular docking. The protein crystal structure was imported into Discovery Studio, and the Prepare Protein module was used to remove water, add hydrogen atom and complement incomplete residues. Active pocket was defined based on the original ligand in the complex, and molecular docking was conducted by CDOCKER algorithm. The root-mean-square-deviation (RMSD) of the initial and re-docked conformations of the original ligand were calculated. RMSD <2 Å indicated that the conformation obtained by docking could reproduce the binding mode of the ligand and receptor, reflecting the reliability of the docking model. Finally, potential antagonists were screened by the constructed docking model. Scoring function of –CDOCKER interaction energy was used to evaluate the binding ability of ligand and receptor.

## Results

### Potential targets of WSYR as an infertility treatment

**FIGURE 1:**
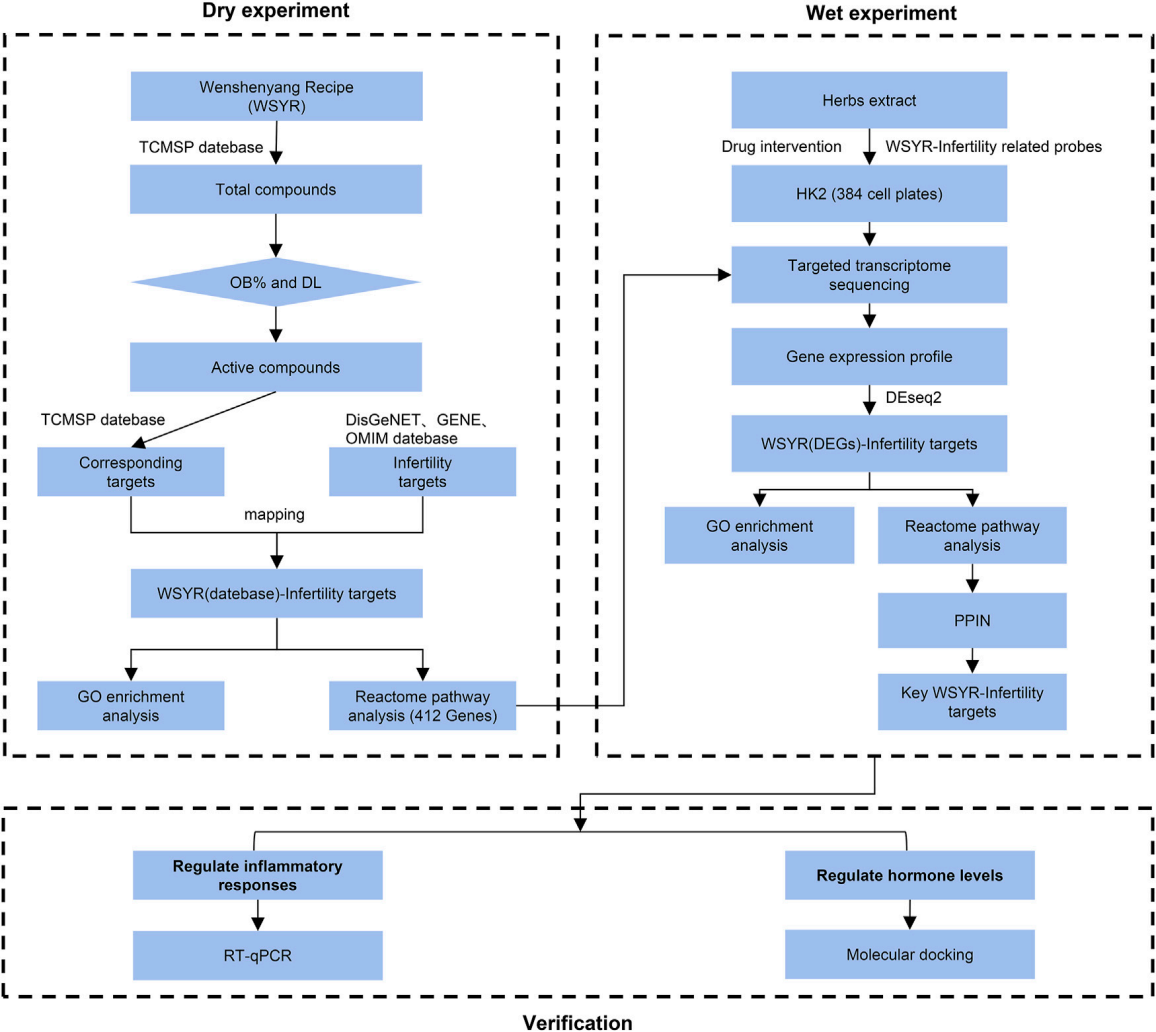
Experimental technical roadmap.

Figure 1 shows the technical roadmap of this study. This study combines a database-based dry experiment and a transcriptome analysis-based wet experiment to jointly analyse the mechanism of action of WSYR in the treatment of infertility.

A total of 75 chemical components for Roucongrong, 130 for Yinyanghuo and 71 for Gusuibu were collected from TCMSP. With a screening criterion of OB ≥ 30% and DL ≥ 0.18, 6 compounds were identified as active compounds for Roucongrong, 23 for Yinyanghuo, 18 for Gusuibu, and 43 for all (Table 1). The 43 WSYR compounds predicted as active compounds for WSYR based on database were used for molecular docking, which were screened by OB ≥ 30% and DL ≥ 0.18 (as listed in Table 1). In addition, 4 compounds were shared by two herbs. For example, luteolin and kaempferol were shared by Yinyanghuo and Gusuibu, β-sitosterol was shared by Roucongrong and Gusuibu, and quercetin was shared by Roucongrong and Yinyanghuo.

**TABLE 1:** Gusuibu, Roucongrong and Yinyanghuo compounds with oral bioavailability (OB) ≥ 30% and drug-likeness (DL) ≥ 0.18 from TCMSP.

NO.	Mol ID	Moleculer name	OB(%)	DL	TCM	NO.	Mol ID	Moleculer name	OB(%)	DL	TCM
1	MOL000449	Stigmasterol	43.83	0.76	GU_SUI_BU	23	MOL004382	Yinyanghuo A	56.96	0.77	YIN_YANG_HUO
2	MOL000492	(+)-catechin	54.83	0.24	GU_SUI_BU	24	MOL004396	CID 12468616	52.31	0.22	YIN_YANG_HUO
3	MOL000569	digallate	61.85	0.26	GU_SUI_BU	25	MOL004386	Yinyanghuo E	51.63	0.55	YIN_YANG_HUO
4	MOL001040	(2 R)-5,7-dihydroxy-2-(4-hydroxyphenyl)chroman-4-one	42.36	0.21	GU_SUI_BU	26	MOL004391	8-prenyl-flavone	48.54	0.25	YIN_YANG_HUO
5	MOL001978	Aureusidin	53.42	0.24	GU_SUI_BU	27	MOL004384	Yinyanghuo C	45.67	0.5	YIN_YANG_HUO
6	MOL002914	Eriodyctiol (flavanone)	41.35	0.24	GU_SUI_BU	28	MOL004373	Anhydroicaritin	45.41	0.44	YIN_YANG_HUO
7	MOL004328	naringenin	59.29	0.21	GU_SUI_BU	29	MOL001645	Linoleyl acetate	42.1	0.2	YIN_YANG_HUO
8	MOL005190	eriodictyol	71.79	0.24	GU_SUI_BU	30	MOL004394	Anhydroicaritin-3-O-alpha-l-rhamnoside	41.58	0.61	YIN_YANG_HUO
9	MOL009061	22-Stigmasten-3-one	39.25	0.76	GU_SUI_BU	31	MOL004425	Icariin	41.58	0.61	YIN_YANG_HUO
10	MOL009063	Cyclolaudenol acetate	41.66	0.79	GU_SUI_BU	32	MOL004380	2,7-Dihydrohomoerysotrine	39.14	0.49	YIN_YANG_HUO
11	MOL009075	cycloartenone	40.57	0.79	GU_SUI_BU	33	MOL003542	8-Isopentenyl-kaempferol	38.04	0.39	YIN_YANG_HUO
12	MOL009076	cyclolaudenol	39.05	0.79	GU_SUI_BU	34	MOL001510	24-epicampesterol	37.58	0.71	YIN_YANG_HUO
13	MOL009078	davallioside A_qt	62.65	0.51	GU_SUI_BU	35	MOL001771	poriferast-5-en-3beta-ol	36.91	0.75	YIN_YANG_HUO
14	MOL009087	marioside_qt	70.79	0.19	GU_SUI_BU	36	MOL000359	sitosterol	36.91	0.75	YIN_YANG_HUO
15	MOL009091	xanthogalenol	41.08	0.32	GU_SUI_BU	37	MOL003044	Chryseriol	35.85	0.27	YIN_YANG_HUO
16	MOL005320	arachidonate	45.57	0.2	ROU_CONG_RONG	38	MOL001792	Liquiritigenin	32.76	0.18	YIN_YANG_HUO
17	MOL005384	suchilactone	57.52	0.56	ROU_CONG_RONG	39	MOL004427	Icariside A7	31.91	0.86	YIN_YANG_HUO
18	MOL008871	Marckine	37.05	0.69	ROU_CONG_RONG	40	MOL000006	luteolin	36.16	0.25	GU_SUI_BU; YIN_YANG_HUO
19	MOL007563	Yangambin	57.53	0.81	ROU_CONG_RONG	41	MOL000098	quercetin	46.43	0.28	ROU_CONG_RONG; YIN_YANG_HUO
20	MOL000622	Magnograndiolide	63.71	0.19	YIN_YANG_HUO	42	MOL000358	beta-sitosterol	36.91	0.75	GU_SUI_BU; ROU_CONG_RONG
21	MOL004367	olivil	62.23	0.41	YIN_YANG_HUO	43	MOL000422	kaempferol	41.88	0.24	GU_SUI_BU; YIN_YANG_HUO
22	MOL004388	CID 12115137	60.64	0.66	YIN_YANG_HUO						

Based on the TCMSP database, 201 potential targets were identified for WSYR, including 110 for Gusuibu, 90 for Roucongrong and 111 for Yinyanghuo. Detailed information about these targets is listed in Supplementary Table S2.

Three databases, OMIM, NCBI-Gene and DisGeNET, were used to search for infertility-related genes. A total of 642 genes were identified, including 246 from OMIM, 448 from NCBI-Gene and 124 from DisGeNET (Supplementary Table S3).

**FIGURE 2:**
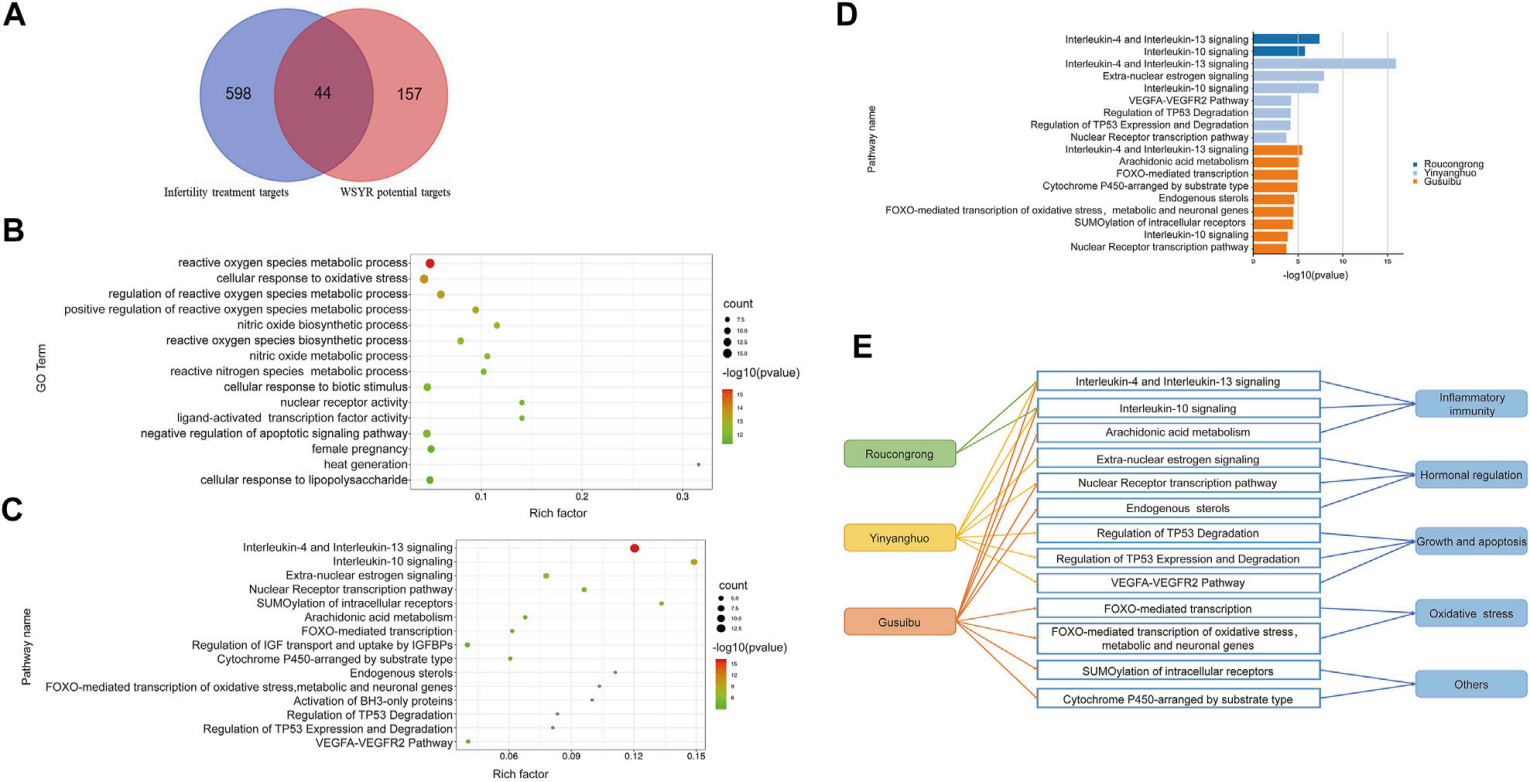
Functional analysis of infertility-related targets of WSYR identified in dry experiments **(A)** Venn diagram of the potential targets in WSYR and the therapeutic targets for infertility **(B)** Bubble chart of GO biological process enrichment analysis of 44 infertility treatment targets in WSYR **(C)** Bubble chart of pathway enrichment analysis of 44 infertility treatment targets in WSYR (*p* value ≤ 10^−3^) **(D)** Pathway enrichment analysis of infertility treatment-related targets of Roucongrong, Yinyanghuo and Gusuibu (*p* value ≤ 10^−3^) **(E)** Comparison of pathways regulated by Roucongrong, Yinyanghuo and Gusuibu.

Of those 642 genes, 22 genes were identified as potential target genes in treating infertility for Roucongrong, 25 for Yinyanghuo and 25 for Gusuibu, 44 in total, as shown in Figure 2A and Table 2.

**TABLE 2:** Database-based infertility treatment targets of Roucongrong, Yinyanghuo and Gusuibu.

NO.	Gene name	Entrez gene	TCM	NO.	UniProt ID	Gene name	TCM	NO.	Gene name	Entrez gene	TCM
1	ADRB1	153	Gusuibu	1	ADRB2	154	Roucongrong	1	ADRB2	154	Yinyanghuo
2	ADRB2	154	Gusuibu	2	AKT1	207	Roucongrong	2	AKT1	207	Yinyanghuo
3	AHR	196	Gusuibu	3	GSTM1	2944	Roucongrong	3	INSR	3643	Yinyanghuo
4	AKT1	207	Gusuibu	4	IL6	3569	Roucongrong	4	NFE2L2	4780	Yinyanghuo
5	APOB	338	Gusuibu	5	INSR	3643	Roucongrong	5	NOS3	4846	Yinyanghuo
6	CAT	847	Gusuibu	6	NFE2L2	4780	Roucongrong	6	PTGS2	5743	Yinyanghuo
7	CYP1A1	1543	Gusuibu	7	NOS3	4846	Roucongrong	7	TGFB1	7040	Yinyanghuo
8	CYP1B1	1545	Gusuibu	8	PGR	5241	Roucongrong	8	PARP1	142	Yinyanghuo
9	CYP19A1	1588	Gusuibu	9	PLAU	5328	Roucongrong	9	AR	367	Yinyanghuo
10	ESR1	2099	Gusuibu	10	PON1	5444	Roucongrong	10	IL10	3586	Yinyanghuo
11	NR3C1	2908	Gusuibu	11	PTGS2	5743	Roucongrong	11	CHEK2	11200	Yinyanghuo
12	GSTM1	2944	Gusuibu	12	TGFB1	7040	Roucongrong	12	AHR	196	Yinyanghuo
13	GSTP1	2950	Gusuibu	13	TNF	7124	Roucongrong	13	ESR1	2099	Yinyanghuo
14	IFNG	3458	Gusuibu	14	PARP1	142	Roucongrong	14	GSTP1	2950	Yinyanghuo
15	IL6	3569	Gusuibu	15	AR	367	Roucongrong	15	IFNG	3458	Yinyanghuo
16	INSR	3643	Gusuibu	16	E2F1	1869	Roucongrong	16	BIRC5	332	Yinyanghuo
17	NFE2L2	4780	Gusuibu	17	HSF1	3297	Roucongrong	17	ESR2	2100	Yinyanghuo
18	NOS3	4846	Gusuibu	18	IGF2	3481	Roucongrong	18	HSPB1	3315	Yinyanghuo
19	PGR	5241	Gusuibu	19	IL10	3586	Roucongrong	19	IL1A	3552	Yinyanghuo
20	PLAU	5328	Gusuibu	20	PTPN1	5770	Roucongrong	20	IL1B	3553	Yinyanghuo
21	PON1	5444	Gusuibu	21	SPP1	6696	Roucongrong	21	MMP2	4313	Yinyanghuo
22	PTGS2	5743	Gusuibu	22	CHEK2	11200	Roucongrong	22	PDE3A	5139	Yinyanghuo
23	SREBF1	6720	Gusuibu					23	CCL2	6347	Yinyanghuo
24	TGFB1	7040	Gusuibu					24	TP53	7157	Yinyanghuo
25	TNF	7124	Gusuibu					25	VEGFA	7422	Yinyanghuo

### Gene ontology and pathway enrichment analysis of infertility-related WSYR targets

There were 44 nonredundant infertility-related target genes in WSYR. To further explore the biological mechanisms of these 44 infertility targets of WSYR, GO biological process (BP) enrichment analysis was carried out. The targets were found to be involved in a variety of biological processes. The top 15 GO BPs are shown in Figure 2B and include the regulation of reactive oxygen species (ROS), the synthesis and metabolism of nitric oxide (NO), cellular response to oxidative stress, nuclear receptor activity, ligand-activated transcription factor activity, negative regulation of apoptosis signaling pathways, female pregnancy, heat generation, and cellular response to lipopolysaccharide.

At low concentrations, ROS participate in physiological regulation as a normal product of aerobic metabolism. However, excessive ROS can cause oxidative stress, which is regarded as related to oocyte maturation, embryonic development, and endometrial translocation (Wan and Qi, 2017). NO is an important free radical species that is important in embryonic development. The activity of NO and NOS is necessary for embryos to initiate the formation of the vasculature, and inhibition of NOS can lead to developmental defects or death of the embryo (Wu et al., 2020; Hitchler and Domann, 2021). Nuclear receptors play a very important role in the regulation of the final stage of ovarian follicle growth. Under the influence of follicle stimulating hormone, nuclear receptors promote the proliferation and differentiation of granulosa cells and the synthesis of steroids (Hughes and Murphy, 2021). WSYR may promote oocyte maturation and embryo development through these biological processes.

Pathway enrichment analysis was performed on the 44 infertility-related target genes of WSYR by ClueGO, and 15 pathways were enriched with a *p* value ≤ 10^−3^, as shown in Figure 2C. Among them, extra-nuclear estrogen signaling, the nuclear receptor transcription pathway, and endogenous sterols are all estrogen-related signaling pathways; regulation of TP53 degradation, regulation of TP53 expression and degradation, and the VEGFA-VEGFR2 pathway are all growth and apoptosis related pathways; interleukin-4 (IL-4) and interleukin-13 (IL-13) signaling, interleukin-10 (IL-10) signaling, and arachidonic acid metabolism are inflammatory signaling pathways; FOXO-mediated transcription, FOXO-mediated transcription of oxidative stress, metabolic and neuronal genes are oxidative stress signaling pathways. Those pathways are closely related to infertility, and involve mechanisms such as sex hormone synthesis and secretion, growth and apoptosis, inflammatory responses, and oxidative stress.

Pathway enrichment analysis was also performed on 22 infertility-related target genes for Roucongrong, 25 for Yinyanghuo and 25 for Gusuibu. As shown in Figure 2D, 2 pathways were enriched for Roucongrong, 7 pathways for Yinyanghuo and 9 pathways for Gusuibu. Therefore, we concluded that WSYR acts on multiple signaling pathways to regulate infertility and that all three constitutive herbs have both common and unique pathways, as shown in Figure 2E. All three herbs regulate inflammatory pathways. Yinyanghuo and Gusuibu both act on hormone regulation pathways. Yinyanghuo acts specially on growth and apoptosis signaling. The unique pathways of Gusuibu include FOXO-mediated transcription, arachidonic acid metabolism and endogenous sterols.

### Construction of a “Herbs-Compounds-Targets-Pathways” network for WSYR

**FIGURE 3:**
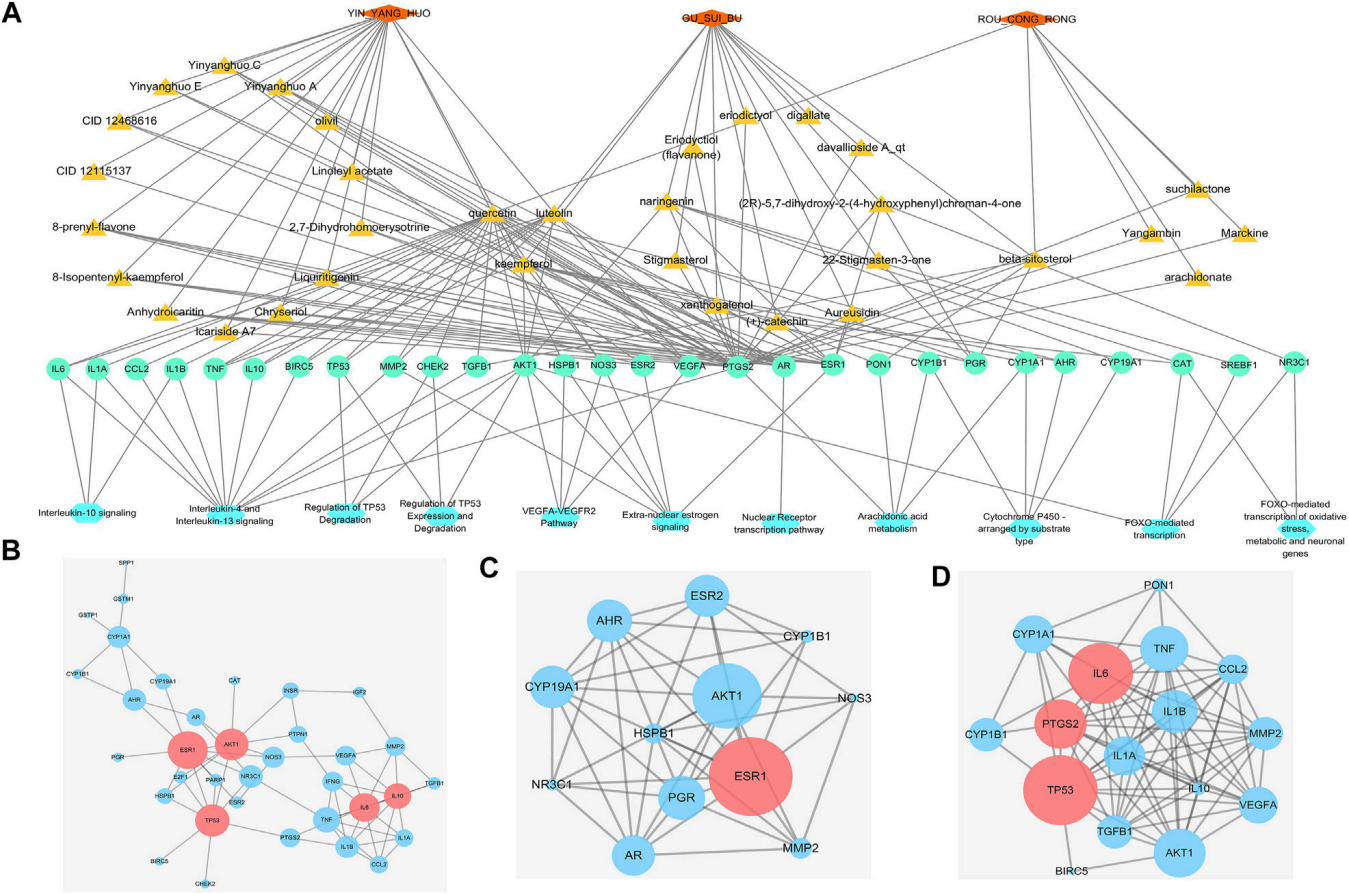
Interactive networks of infertility treatment targets based on databases **(A)**“Herbs-Compounds-Targets-Pathways” network. Orange diamond: TCM; dark yellow triangle: active compound; green circle: gene; blue hexagon: pathway **(B)** Protein–protein interaction network (PPIN) of 44 WSYR infertility therapeutic targets. Dark pink circle: key targets; blue circle: other targets **(C)** Hormone-related PPIN based on database targets. Dark pink circle: key targets; blue circle: other targets **(D)** Inflammatory immune-regulated PPIN based on database targets. Dark pink circle: key targets; blue circle: other targets.

As shown in Figure 3A, a “Herbs-Compounds-Targets-Pathways” network was constructed to show the overall function of WSYR and its constitutive herbs.

Yinyanghuo A, Yinyanghuo B and Yinyanghuo C, representative compounds of Yinyanghuo, play immune inflammation regulatory and sex hormone regulatory roles in the treatment of infertility. The above three compounds all act on prostaglandin G/H synthase 2 (PTGS2) and androgen receptor (AR). Yinyanghuo A acts on ESR1, and Yinyanghuo B acts on ESR2. PTGS2 exerts inflammatory immune regulation by affecting IL-4 and IL-13 signaling, IL-10 signaling, and arachidonic acid metabolism. It has been reported that PTGS2 is closely associated with ovulation failure and implantation disorders in infertility (Szczuko et al., 2020). AR, ESR1 and ESR2 regulate the nuclear receptor transcription pathway, and ESR1 and ESR2 regulate extra-nuclear estrogen signaling to play a hormonal regulatory role.

As components of Gusuibu (+)-catechin and naringenin play a role in regulating oxidative stress in the treatment of infertility. Both (+)-catechin and naringenin can act on catalase (CAT). CAT is an enzyme involved in oxidative stress detoxification. It protects the human body from oxidative stress by regulating FOXO-mediated transcription and FOXO-mediated transcription of oxidative stress.

The Roucongrong component quercetin plays a role in regulating inflammation immunity in infertility. Quercetin acts on IL-10, IL-1α, IL-1β, IL-6, TNF, CCL2 and other inflammatory factors and chemokines and regulates IL-4 and IL-13 signaling and IL-10 signaling.

### Construction of protein-protein interaction networks of WSYR based on dry experiments

A total of 44 WSYR infertility therapeutic targets were used to construct a PPIN (confidence level >0.9) and to calculate the degree, betweenness and closeness of each node in the network. The larger the three parameter values, the more critical are the targets in the network. The top 5 nodes with the highest sum of degree, betweenness and closeness were proposed as key targets of WSYR, including ESR1, TP53, AKT1, IL-6, and IL-10 (Figure 3B and Supplementary Table S4-A).

We constructed a hormone regulation-related PPIN (confidence level >0.4) involving the three pathways of extra-nuclear estrogen signaling, nuclear receptor transcription pathway, and endogenous sterols and calculated the degree, betweenness and closeness of each node in the network. ESR1 was the most critical target (Figure 3C and Supplementary Table S4-B).

In addition, we constructed an immune inflammation-related PPIN (confidence level >0.4) involving interleukin-4 and interleukin-13 signaling, interleukin-10 signaling, and arachidonic acid metabolism, and calculated the degree, betweenness and closeness of each node in the network. The key targets included TP53, IL-6, and PTGS2 (Figure 3D and Supplementary Table S4-C).

### Differentially expressed genes identified by transcriptome analysis in HK-2 cells

Due to the incomplete and unsystematic nature of network pharmacology, the predicted results may not fully reflect the actual mechanism of WSYR. The application of transcriptome analysis can identify differentially expressed genes in response to TCM treatment and complement the results from network pharmacology.

There is an old saying in Chinese medicine: the kidney stores the essence, which is the foundation of reproduction. Deficiency of kidney qi and inadequacy of Tiangui will result in insufficient production of fertilization-competent sperm, leading to infertility. Deficiency of kidney yang, inability to warm semen and warm uterus, can cause cold semen and cold uterus, thereby causing infertility. Based on this, transcriptome analysis was performed on HK-2 human renal epithelial cells to examine the transcriptional regulation of WSYR in infertility-related pathways.

According to the results above, 15 pathways with *p* values less than 10^−3^ are the most important in the treatment of infertility by WSYR. Therefore, the 412 genes in these 15 pathways were subjected to transcriptome analysis in HK-2 cells treated with Roucongrong, Yinyanghuo and Gusuibu.

**FIGURE 4:**
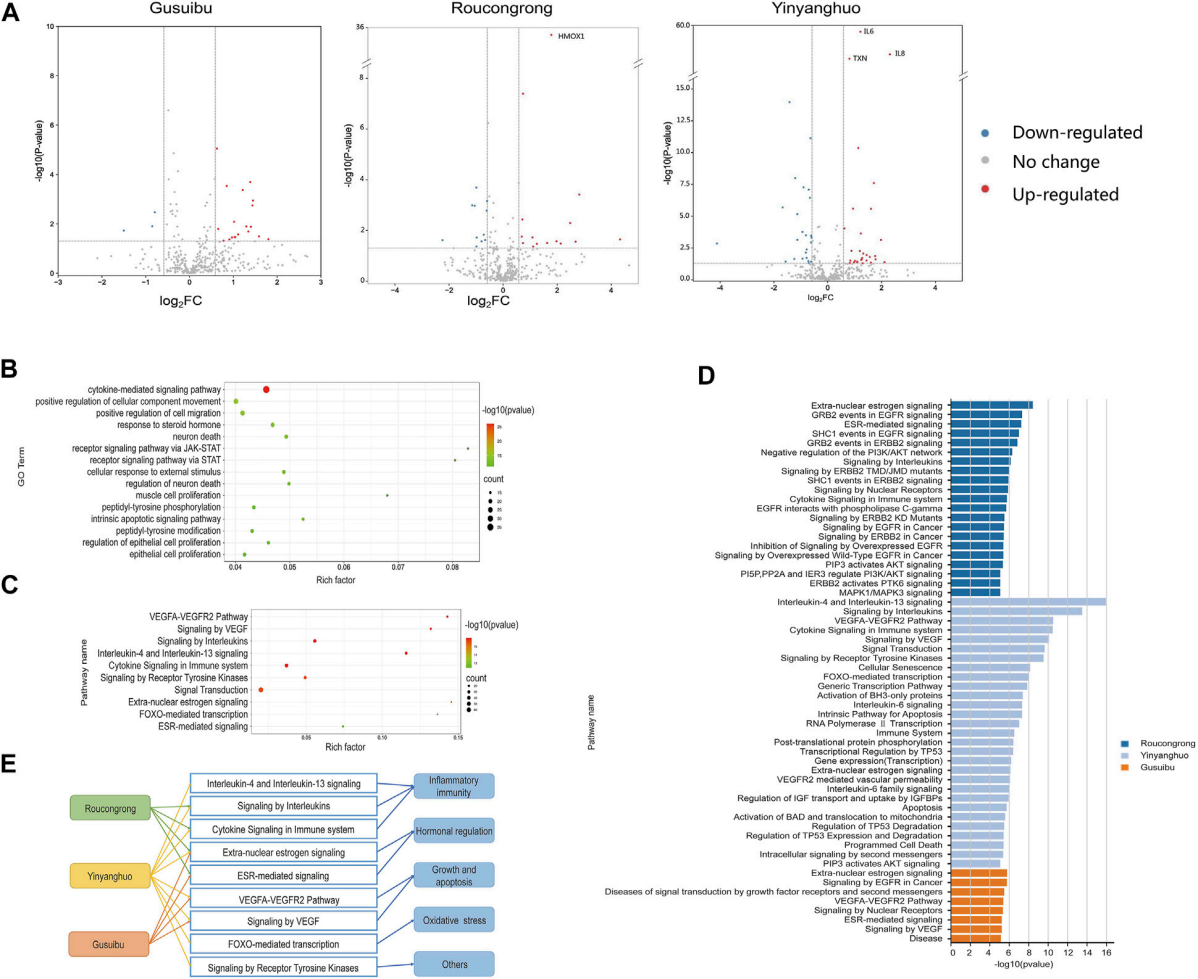
Functional analysis of WSYR DEGs based on transcriptome analysis **(A)** Volcano map of DEGs of Roucongrong, Yinyanghuo and Gusuibu. Red dots: upregulated genes; green dots: downregulated genes; black dots: unchanged genes **(B)** Bubble chart of GO biological processes enriched among the 92 DEGs related to WSYR (top 15) **(C)** Bubble chart of pathways enriched among the 92 DEGs related to WSYR (top 10) **(D)** Pathway enrichment analysis of DEGs in Roucongrong, Yinyanghuo and Gusuibu (*p* value ≤ 10^−5^) **(E)** Comparison of pathways regulated by Roucongrong, Yinyanghuo and Gusuibu.

As shown in the volcano map (Figure 4A), 26 DEGs were identified in Roucongrong, including 15 upregulated genes and 11 downregulated genes; 54 DEGs were identified in Yinyanghuo, including 30 upregulated genes and 24 downregulated genes; and 22 DEGs were identified in Gusuibu, including 19 upregulated genes and 3 downregulated genes (FC > 1.5 or <0.67 and *p* < 0.05). In conclusion, a total of 92 DEGs (Table 3) were identified for WSYR based on transcriptome analysis.

**TABLE 3:** 92 DEGs of WSYR. Including 22 gusuibu DEGs, 26 RoucongrongDEGs and 54 yinyanghuo DEGs.

NO.	Gene symbol	Entrez gene	log2FC	*p* Value	Classification	NO.	Gene symbol	Entrez gene	log2FC	*p* Value	Classification	NO.	Gene symbol	Entrez gene	log2FC	*p* Value	Classification
1	EREG	2069	0.626	8.91E-06	Gusuibu	13	FOXO4	4303	1.087	1.88E-02	Roucongrong	21	NCK1	4690	−0.601	4.70E-04	Yinyanghuo
2	PTK2B	2185	1.386	2.03E-04	Gusuibu	14	CYBA	1535	−0.977	1.90E-02	Roucongrong	22	IGFBP7	3490	−1.128	7.29E-04	Yinyanghuo
3	VCAN	1462	0.849	2.91E-04	Gusuibu	15	GNG3	2785	4.330	2.25E-02	Roucongrong	23	CXCL1	2919	1.978	7.35E-04	Yinyanghuo
4	CAV2	858	1.214	4.29E-04	Gusuibu	16	TYK2	7297	−0.663	2.41E-02	Roucongrong	24	WASF1	8936	−0.623	1.15E-03	Yinyanghuo
5	PLCG1	5335	1.448	1.14E-03	Gusuibu	17	ESR2	2100	−2.243	2.45E-02	Roucongrong	25	RASA1	5921	−4.118	1.41E-03	Yinyanghuo
6	PIM1	5292	1.435	1.80E-03	Gusuibu	18	MMP7	4316	1.977	2.68E-02	Roucongrong	26	TP53	7157	−0.781	4.20E-03	Yinyanghuo
7	STAT3	6774	−0.792	3.42E-03	Gusuibu	19	BCL2L1	598	−0.797	2.69E-02	Roucongrong	27	NR1H2	7376	0.882	5.24E-03	Yinyanghuo
8	TXNIP	10628	1.017	8.30E-03	Gusuibu	20	IL1R1	3554	2.682	2.78E-02	Roucongrong	28	VCAM1	7412	1.191	5.48E-03	Yinyanghuo
9	GADD45 A	1647	−0.851	1.25E-02	Gusuibu	21	EGF	1950	1.629	3.13E-02	Roucongrong	29	CALM1	801	−0.814	7.22E-03	Yinyanghuo
10	CYP26C1	340665	1.296	1.27E-02	Gusuibu	22	HSPA8	3312	0.736	3.14E-02	Roucongrong	30	ARNT	405	1.323	8.66E-03	Yinyanghuo
11	KDR	3791	1.400	1.32E-02	Gusuibu	23	NR2C2	7182	2.133	3.32E-02	Roucongrong	31	PPARG	5468	1.447	1.14E-02	Yinyanghuo
12	GNAI1	2770	0.655	1.62E-02	Gusuibu	24	SIRT3	23410	1.250	3.41E-02	Roucongrong	32	NR4A2	4929	1.762	1.34E-02	Yinyanghuo
13	ARNT	405	−1.500	1.88E-02	Gusuibu	25	PIAS1	8554	−0.986	4.27E-02	Roucongrong	33	IL6R	3570	1.563	1.60E-02	Yinyanghuo
14	MARC1	64757	1.342	2.03E-02	Gusuibu	26	BCL6	604	1.112	4.30E-02	Roucongrong	34	SGK1	6446	−0.785	1.87E-02	Yinyanghuo
15	GNGT2	2793	1.110	2.70E-02	Gusuibu	1	IL6	3569	1.382	1.01E-55	Yinyanghuo	35	ATM	472	1.279	1.89E-02	Yinyanghuo
16	POMC	5443	1.586	3.24E-02	Gusuibu	2	IL8	3576	2.331	5.35E-27	Yinyanghuo	36	YWHAH	7533	−0.937	2.20E-02	Yinyanghuo
17	BMF	90427	1.028	3.42E-02	Gusuibu	3	TXN	7295	0.615	1.92E-24	Yinyanghuo	37	CYP4V2	285440	−1.242	2.32E-02	Yinyanghuo
18	KAT2B	8850	1.048	3.43E-02	Gusuibu	4	FN1	2335	−1.420	1.06E-14	Yinyanghuo	38	ROCK1	6093	1.777	2.42E-02	Yinyanghuo
19	F5	2153	0.969	3.53E-02	Gusuibu	5	HSP90B1	7184	−0.640	7.51E-12	Yinyanghuo	39	F5	2153	1.260	2.57E-02	Yinyanghuo
20	SHC2	25759	1.798	4.17E-02	Gusuibu	6	HMOX1	3162	1.134	4.38E-11	Yinyanghuo	40	IL23 A	51561	1.242	2.65E-02	Yinyanghuo
21	THRB	7068	0.909	4.24E-02	Gusuibu	7	STAT1	6772	−1.208	9.97E-09	Yinyanghuo	41	FOXO4	4303	1.443	3.10E-02	Yinyanghuo
22	PIK3R1	5295	0.778	4.81E-02	Gusuibu	8	CXCL2	2920	1.716	2.47E-08	Yinyanghuo	42	CYP2S1	29785	0.841	3.11E-02	Yinyanghuo
1	HMOX1	3162	1.873	2.87E-36	Roucongrong	9	CAV1	857	−0.899	5.29E-08	Yinyanghuo	43	ITGB2	3689	1.011	3.28E-02	Yinyanghuo
2	EREG	2069	0.795	4.12E-08	Roucongrong	10	CDKN2A	1029	−0.705	8.18E-08	Yinyanghuo	44	HDAC1	3065	−0.721	3.32E-02	Yinyanghuo
3	VCAN	1462	−0.990	2.03E-04	Roucongrong	11	SDC2	6383	−0.660	3.56E-07	Yinyanghuo	45	SOCS5	9655	1.091	3.64E-02	Yinyanghuo
4	TNFRSF1A	7132	2.819	3.81E-04	Roucongrong	12	VCAN	1462	−1.675	2.01E-06	Yinyanghuo	46	PAK3	5063	−1.565	3.65E-02	Yinyanghuo
5	EGFR	1956	−0.593	7.01E-04	Roucongrong	13	EREG	2069	0.942	2.60E-06	Yinyanghuo	47	LAMC1	3915	−0.622	3.72E-02	Yinyanghuo
6	CYP4V2	285440	−1.145	1.02E-03	Roucongrong	14	CDKN1A	1026	1.609	2.64E-06	Yinyanghuo	48	FOS	2353	2.111	4.13E-02	Yinyanghuo
7	SOD2	6648	−1.052	1.05E-03	Roucongrong	15	ITGAV	3685	−1.131	6.93E-06	Yinyanghuo	49	PPARGC1A	10891	1.096	4.16E-02	Yinyanghuo
8	MED1	5469	−0.609	1.68E-03	Roucongrong	16	IL18	3606	0.619	9.06E-05	Yinyanghuo	50	AKT2	208	0.975	4.24E-02	Yinyanghuo
9	SUMO2	6613	0.713	3.71E-03	Roucongrong	17	LCN2	3934	−0.940	1.70E-04	Yinyanghuo	51	MMP7	4316	1.605	4.53E-02	Yinyanghuo
10	HRAS	3265	2.482	5.12E-03	Roucongrong	18	BAD	572	1.229	2.24E-04	Yinyanghuo	52	JUNB	3726	0.822	4.71E-02	Yinyanghuo
11	THEM4	117145	−0.715	1.49E-02	Roucongrong	19	CTNND1	1500	−0.810	3.16E-04	Yinyanghuo	53	PTK2	5747	−0.704	4.77E-02	Yinyanghuo
12	PPP2CA	5515	0.691	1.77E-02	Roucongrong	20	TP53BP2	7159	−0.619	3.40E-04	Yinyanghuo	54	SFN	2810	1.300	4.94E-02	Yinyanghuo

### Gene ontology and pathway enrichment analysis of WSYR DEGs

To further explore the biological mechanism of these 92 DEGs in WSYR, GO BP enrichment analysis was carried out. As shown in Figure 4B, the DEGs were involved in a variety of biological processes, including regulation of cytokine-mediated signaling pathways, cell movement and migration, steroid hormone response, JAK-STAT receptor signaling pathways, neuronal death, intrinsic apoptosis signaling pathways, peptidyl-tyrosine phosphorylation modification, and proliferation of muscle and epithelial cells.

When inflammation occurs, cells such as leukocytes, fibroblasts, endothelial cells, and haematopoietic cells are induced to produce chemokines by IL-1, TNF and other cytokines and are guided by chemokines to migrate to specific tissues (Borrelli et al., 2013). Studies have found that the chemokines CXCL1 and CXCL13 are abnormally expressed in female infertility patients with chronic endometritis (Kitaya and Yasuo, 2010). Sex steroid hormones are essential elements that affect reproduction. They affect follicle formation, endometrial development, ovulation, implantation and other developmental events (Reichman and Rosenwaks, 2017) that are critical for female pregnancy. In addition, members of the JAK-STAT pathway, TYK2, STAT1 and STAT4, have been shown to be active in human sperm, indicating that defects in the JAK-STAT pathway in sperm may be related to male infertility (D'Cruz et al., 2001).

Similarly, many pathways were enriched by the 92 DEGs of WSYR; the top 10 pathways are shown in Figure 4C. Among them, extra-nuclear estrogen signaling and ESR-mediated signaling are both estrogen-related signaling pathways; the VEGFA-VEGFR2 pathway and signaling by VEGF are both growth- and apoptosis-related pathways; Interleukin-4 and interleukin-13 signaling, signaling by interleukins and cytokine signaling in immune system are inflammatory immune signaling pathways; and FOXO-mediated transcription is an oxidative stress signaling pathway. These results are consistent with those of database-based network pharmacology.

Pathway enrichment analysis of the DEGs of the three herbs was also carried out. The total numbers of enriched pathways with *p* value **≤** 10^−5^ were 21 for Roucongrong, 29 for Yinyanghuo, and 8 for Gusuibu (Figure 4D). WSYR acts on a variety of signaling pathways to regulate infertility, and the three herbs have both common and unique pathways (Figure 4E). For example, Roucongrong plays a role in inflammatory immune and hormone regulation, and Gusuibu plays a role in angiogenesis and hormone regulation. In addition to regulating inflammatory responses, angiogenesis and hormone levels, Yinyanghuo plays a unique role in the regulation of oxidative stress and receptor tyrosine kinases.

### Construction of a “herbs-degs-pathways” network for WSYR based on transcriptome analysis

**FIGURE 5:**
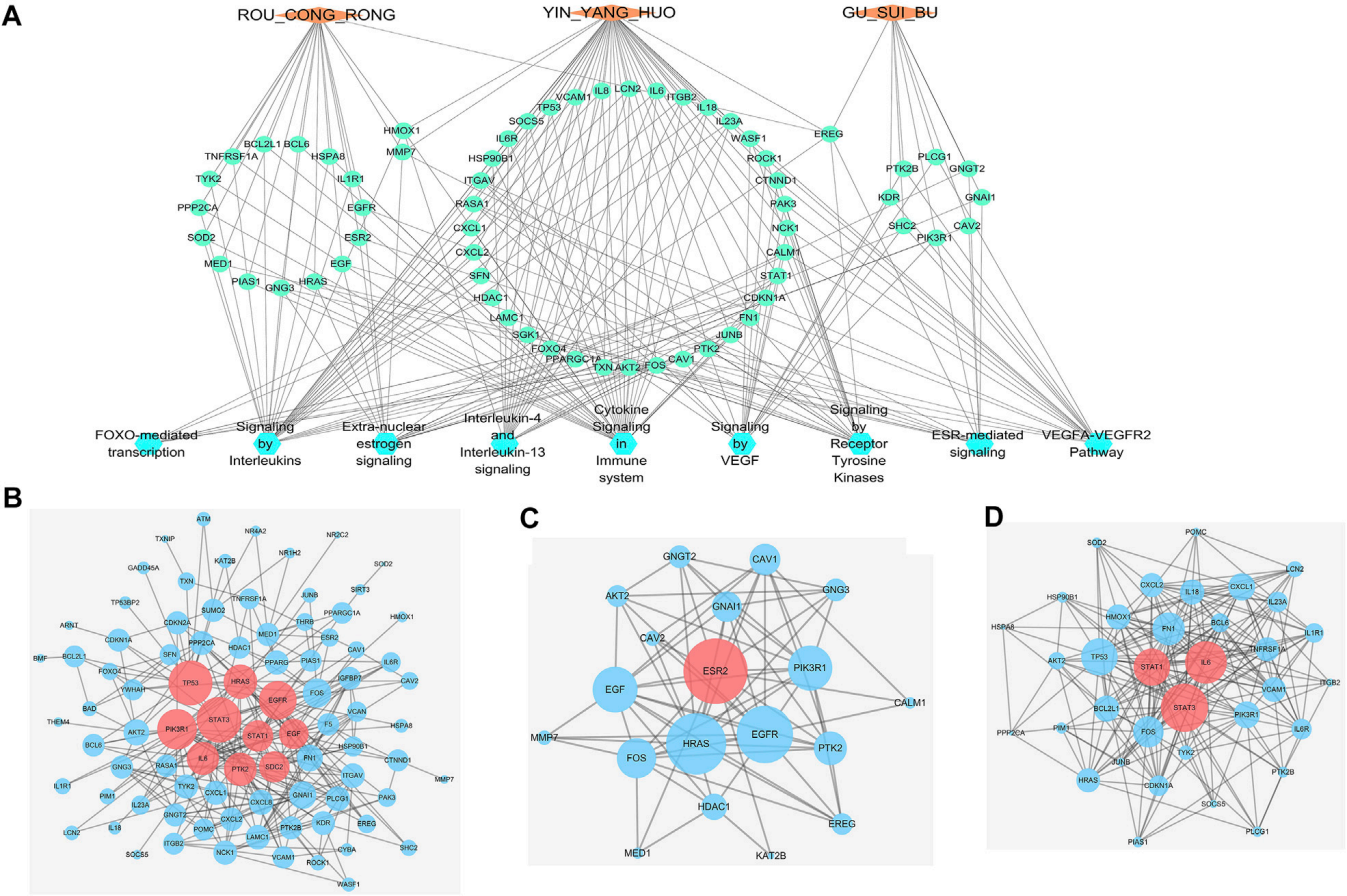
Interactive networks of WSYR based on DEGs **(A)** “Herbs-DEGs-Pathways” network. Orange diamond: TCM; green circle: gene; blue circle: pathway **(B)** Protein—protein interaction network (PPIN) of all DEGs from transcriptome sequencing. Dark pink circle: key targets; blue circle: other targets **(C)** Hormone-related PPIN based on DEGs.Dark pink circle: key targets; blue circle: other targets **(D)** Inflammatory immune-regulated PPIN based on DEGs.Dark pink circle: key targets; blue circle: other targets.

Based on the results of transcriptome analysis, we drew a “Herbs-DEGs-Pathways” diagram to show the overall function of WSYR and its constituent herbs, as shown in Figure 5A.

Proepiregulin (EREG) is coregulated by Roucongrong, Yinyanghuo and Gusuibu and acts on three signaling pathways: extra-nuclear estrogen signaling, ESR-mediated signaling and signaling by receptor tyrosine kinases. EREG is a member of the epidermal growth factor family, which can regulate angiogenesis and remodelling to promote tissue repair, wound healing and oocyte maturation (Riese and Cullum, 2014). Haem oxygenase 1 (HMOX1) is jointly regulated by Roucongrong and Yinyanghuo and acts on three signaling pathways: IL-4 and IL-13 signaling, signaling by interleukins and cytokine signaling in the immune system. HMOX1 catalyses the degradation of haem to produce carbon monoxide (CO), iron and biliverdin-IXα. The HMOX1/CO system has been found to exert cytoprotective effects on damaged organs and in animal models by regulating inflammation and apoptosis (Ryter, 2019). MMP7 is jointly regulated by Roucongrong and Yinyanghuo and acts on three signaling pathways: extranuclear estrogen signaling, ESR-mediated signaling and signal transduction. MMPs can degrade the extracellular matrix (ECM), affect the migration of germ cells, affect the spermatogenesis pathway, and play important roles in spermatogenesis and semen quality (Warinrak et al., 2015).

### Construction of PPINs of WSYR based on transcriptome analysis

To identify the key targets of WSYR, a PPIN was constructed based on the 92 DEGs obtained by transcriptome analysis (confidence level >0.9), and 86 nodes are illustrated in Figure 5B and Supplementary Table S5-A. The degree, betweenness and closeness of each node in the network were calculated. The results showed that the key targets included STAT3, TP53, PIK3R1, EGFR, HRAS, PTK2, IL6, STAT1, SDC2 and EGF.

To further identify the key targets of WSYR in hormones, 19 DEGs (confidence level >0.4) corresponding to the two hormone-related pathways of extra-nuclear estrogen signaling and ESR-mediated signaling in Figure 4E were selected to construct a PPIN. Nineteen nodes are shown in Figure 5C and Supplementary Table S5-B. Additionally, the degree, betweenness and closeness of each node in the network was calculated. The results showed that the most critical target of hormone regulation is ESR2.

To further identify the key targets of WSYR in immune inflammation, 35 DEGs (confidence level >0.4) corresponding to the three inflammatory immune-related pathways interleukin-4 and interleukin-13 signaling, signaling by interleukins, and cytokine signaling in the immune system, as shown in Figure 4E, were selected to construct a PPIN. Thirty-five nodes are shown in Figure 5D and Supplementary Table S5-C. The degree, betweenness and closeness of each node in the network are calculated. The results showed that the key targets of WSYR involved in inflammatory responses included STAT3, IL6 and STAT1.

Based on the dry experiments and wet experiments, we have found that WSYR mainly plays a role in hormone regulation and inflammatory responses. In the next step, we plan to use RT-qPCR to verify the regulatory effect of WSYR on key genes involved in inflammation and to perform molecular docking to further identify the active compounds present in WSYR and validate the interaction between compounds and potential targets of WSYR.

### RT-qPCR validation of WSYR in regulating inflammatory responses

To verify the anti-inflammatory effect of WSYR, we examined target genes in inflammatory responses by RT-qPCR. In this study, IL-4 and IL-10 enriched by the inflammatory pathway were selected for RT-qPCR validation in SK-OV-3 cells. IL-4 and IL-10 are classic anti-inflammatory cytokines that limit excessive tissue destruction caused by inflammation.

**FIGURE 6:**
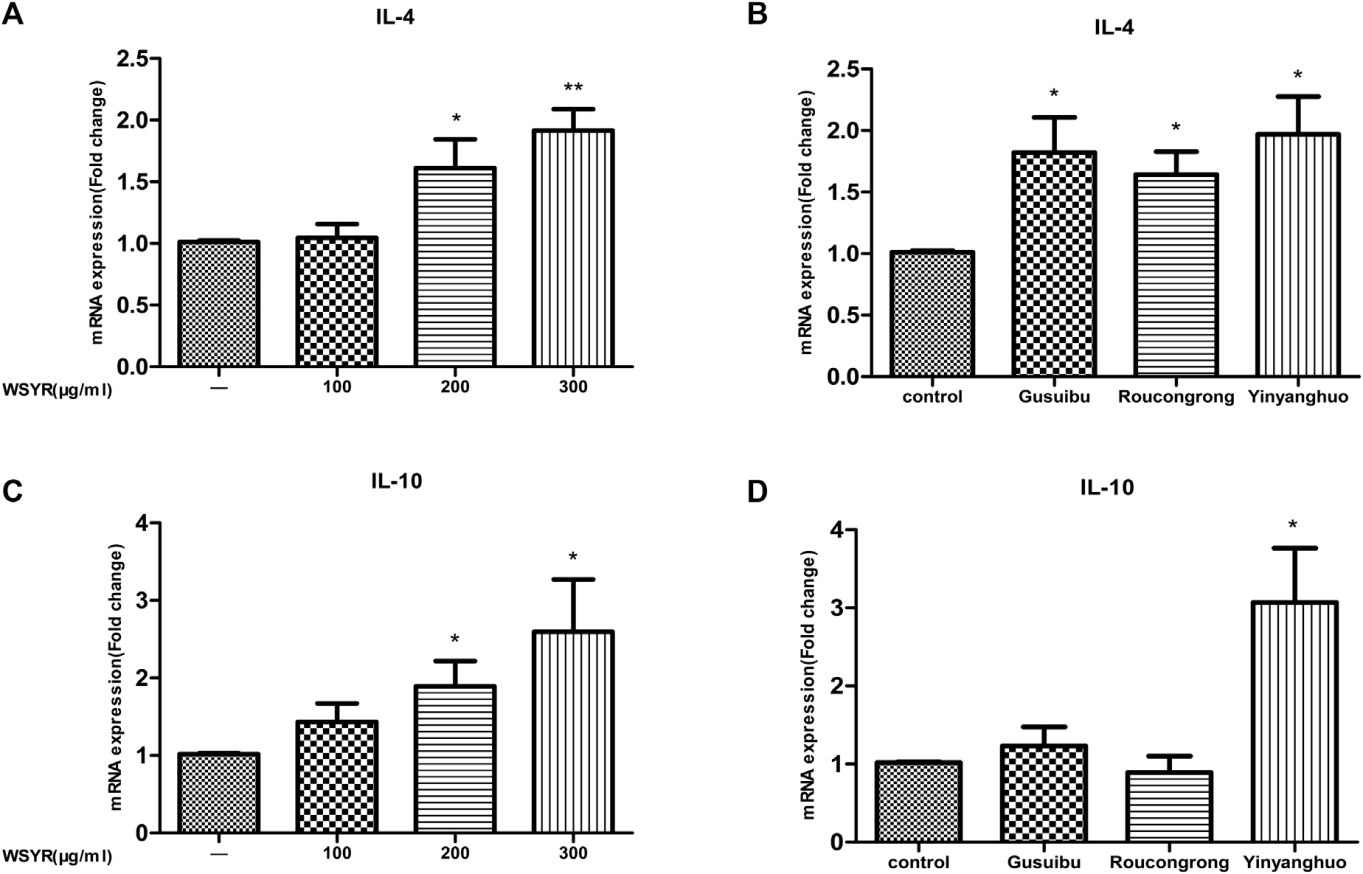
Action of WSYR and its herbs Gusuibu, Roucongrong, Yinyanghuo on SK-OV-3 cells. SK-OV-3 cells were treated with WSYR (100, 200, 300 μg/ml), Gusuibu (200 μg/ml), Roucongrong (200 μg/ml), Yinyanghuo (200 μg/ml) for 24 h. The mRNA expression of *IL-4* **(A,B)**, *IL-10* **(C,D)** was analyzed by qPCR. *p < 0.05 and **p < 0.01 indicate statistical significance compared to control.

The experimental results showed that WSYR could play an anti-inflammatory role by upregulating the expression of *IL-4* and *IL-10* (Figure 6A and Figure 6C). Specifically, Gusuibu and Roucongrong can upregulate the expression of *IL-4*, and Yinyanghuo can upregulate the expression of *IL-4* and *IL10* to exert anti-inflammatory effects (Figure 6B and Figure 6D).

### Construction of ESR1 and ESR2 molecular docking models and drug screening

Studies have reported that estrogen receptors are important for maintaining follicle, oocyte growth and ovulation function (Tang et al., 2019). Our results suggested that WSYR treats infertility by regulating estrogen receptors, but the specific mechanism of action is still unclear. The molecular docking model of ESR1 and ESR2 was constructed, and the compounds of WSYR were evaluated by docking.

**FIGURE 7:**
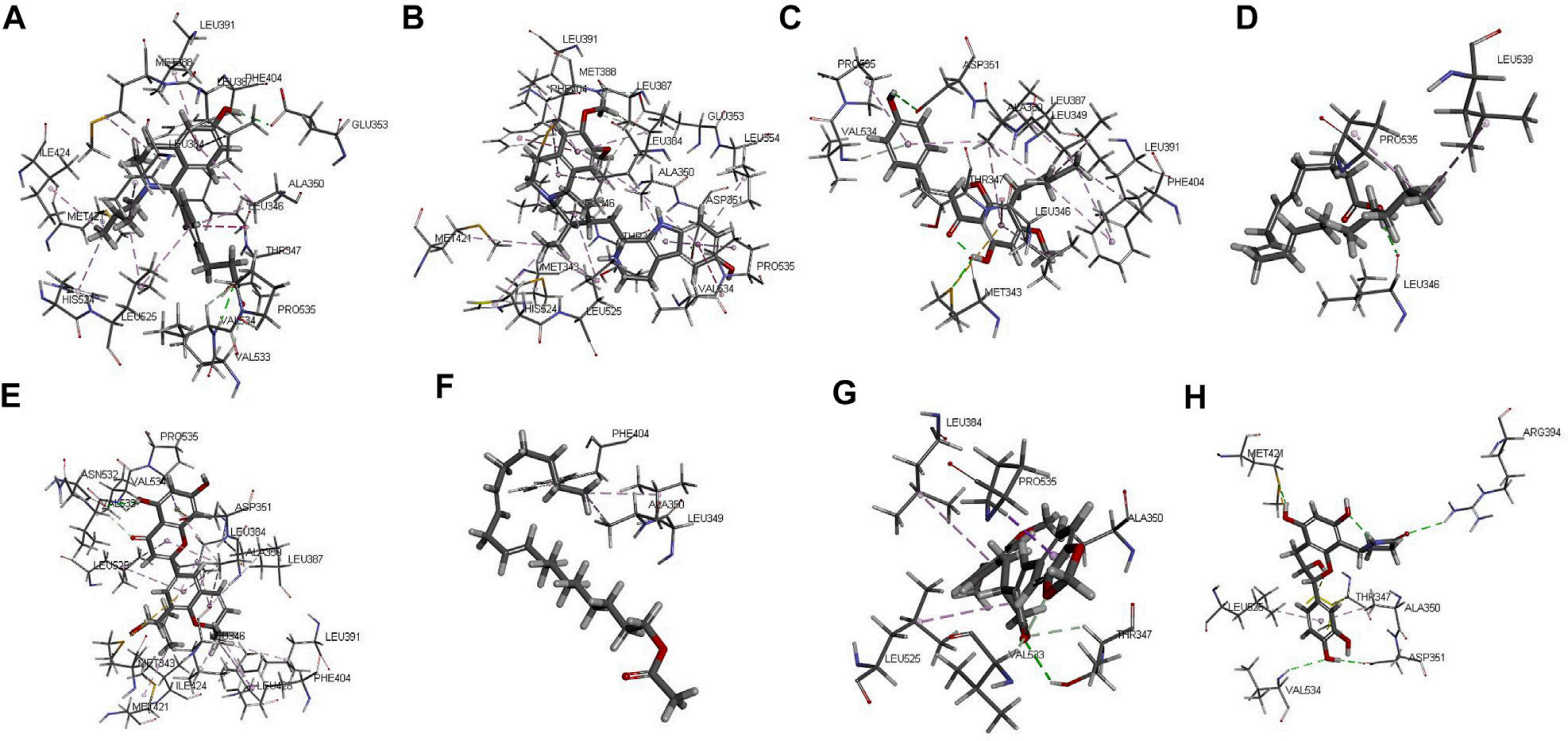
The 3D interaction of the original ligand GQD **(A)**, marckine **(B)**, xanthogalenol **(C)**, arachidonate **(D)** and yinyanghuo A **(E)**, linoleyl acetate **(F)**, suchilactone **(G)** and davallioside A_qt **(H)** with ESR1.

The protein crystal complex (pdb id: 5FQP) was selected as the docking target of ESR1. The original ligand ESR1 antagonist (Scott et al., 2015) was used as the positive control. Its -cdocker interaction energy (-CIE) score is 65.414, RMSD is 0.3557, pocket radius is 8.522, and pocket site is (14.230, 22.133, 65.663). The positive drug score (65.414) was set as the threshold, and the drug score value > threshold×75% as the standard for the drug to have a good affinity with ESR1. The molecular docking results of 43 WSYR compounds with ESR1 are shown in Table 4. Finally, the following components have strong affinity with ESR1: marckine (54.5303), xanthogalenol (53.776), arachidonate (51.688), yinyanghuo A (51.0526), linoleyl acetate (50.8283), suchilactone (50.5918), and davallioside A_qt (49.7764), and the result is shown in Figure 7.

**TABLE 4:** ESR1 molecular docking results.

NO.	ID	-CDOCKER_INTERACTION_ENERGY	Moleculer name	TCM
1	original ligand	65.414	GQD	—
2	MOL008871	54.530	Marckine	Roucongrong
3	MOL009091	53.776	Xanthogalenol	Gusuibu
4	MOL005320	51.688	Arachidonate	Roucongrong
5	MOL004382	51.053	Yinyanghuo A	Yinyanghuo
6	MOL001645	50.828	Linoleyl acetate	Yinyanghuo
7	MOL005384	50.592	Suchilactone	Roucongrong
8	MOL009078	49.776	davallioside A_qt	Gusuibu
9	MOL004367	47.811	Olivil	Yinyanghuo
10	MOL007563	47.178	Yangambin	Roucongrong
11	MOL004396	46.845	CID 12468616	Yinyanghuo
12	MOL003542	46.101	8-Isopentenyl-kaempferol	Yinyanghuo
13	MOL000569	45.932	Digallate	Gusuibu
14	MOL005190	44.156	Eriodictyol	Gusuibu
15	MOL000492	43.466	(+)-catechin	Gusuibu
16	MOL004373	43.293	Anhydroicaritin	Yinyanghuo
17	MOL001978	43.188	Aureusidin	Gusuibu
18	MOL004384	42.640	Yinyanghuo C	Yinyanghuo
19	MOL000098	42.464	Quercetin	Roucongrong, Yinyanghuo
20	MOL001040	42.130	(2 R)-5,7-dihydroxy-2-(4-hydroxyphenyl)chroman-4-one	Gusuibu
21	MOL004386	41.695	Yinyanghuo E	Yinyanghuo
22	MOL004391	40.476	8-prenyl-flavone	Yinyanghuo
23	MOL003044	39.899	Chryseriol	Yinyanghuo
24	MOL002914	39.767	Eriodyctiol (flavanone)	Gusuibu
25	MOL001792	39.745	Liquiritigenin	Yinyanghuo
26	MOL009087	39.734	marioside_qt	Gusuibu
27	MOL000006	39.021	Luteolin	Gusuibu, Yinyanghuo
28	MOL004380	38.627	2,7-Dihydrohomoerysotrine	Yinyanghuo
29	MOL004328	38.601	Naringenin	Gusuibu
30	MOL000422	38.113	Kaempferol	Gusuibu, Yinyanghuo
31	MOL004427	36.341	Icariside A7	Yinyanghuo
32	MOL000622	33.682	Magnograndiolide	Yinyanghuo
33	MOL009061	30.159	22-Stigmasten-3-one	Gusuibu
34	MOL004388	25.106	CID 12115137	Yinyanghuo
35	MOL001771	17.943	poriferast-5-en-3beta-ol	Yinyanghuo
36	MOL009075	16.345	cycloartenone	Gusuibu
37	MOL001510	15.266	24-epicampesterol	Yinyanghuo
38	MOL000449	11.704	Stigmasterol	Gusuibu
39	MOL000359	8.864	sitosterol	Yinyanghuo
40	MOL009076	−0.891	cyclolaudenol	Gusuibu
41	MOL000358	−8.363	beta-sitosterol	Gusuibu, Roucongrong
42	MOL004394	No refined poses	Anhydroicaritin-3-O-alpha-l-rhamnoside	Yinyanghuo
43	MOL004425	No refined poses	Icariin	Yinyanghuo
44	MOL009063	No refined poses	Cyclolaudenol acetate	Gusuibu

**FIGURE 8:**
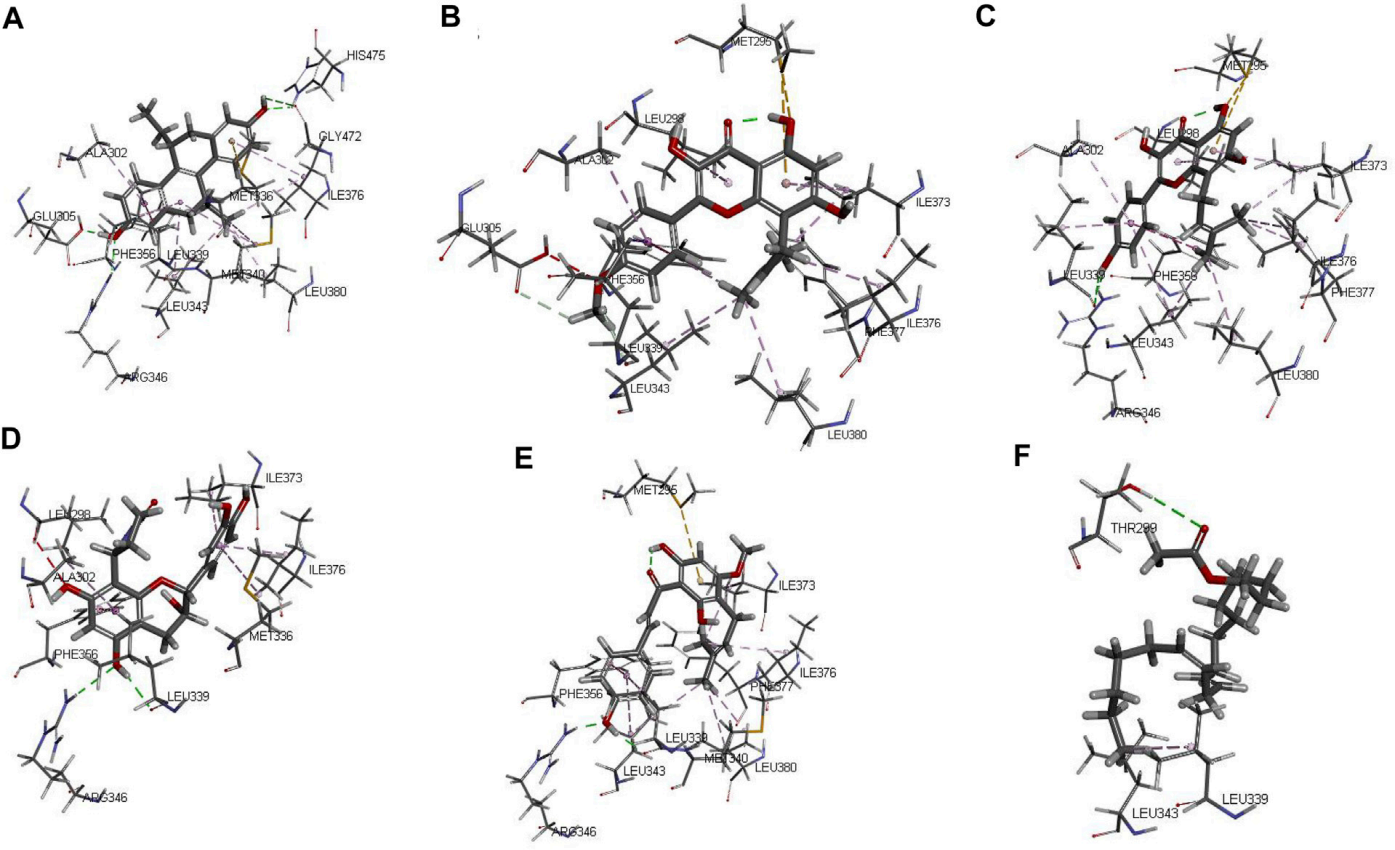
The 3D interaction of the original ligand THC **(A)**, anhydroicaritin **(B)**, 8-isopentenyl-kaempferol **(C)**, davallioside A_qt **(D)**, xanthogalenol **(E)** and linoleyl acetate **(F)** with ESR2.

The protein crystal complex 1L2J (Shiau et al., 2002) was selected as the docking target of ESR2. The original ligand ESR2 antagonist was used as the positive control. Its -cdocker interaction energy score is 52.8724, RMSD is 0.6922, pocket radius is 7.574, and pocket site is (32.212, 82.073, -11.407). The positive drug score (52.8724) was set as the threshold, and the drug score value > threshold×90% was set as the standard for the drug to have a good affinity with ESR2. The molecular docking results of 43 WSYR compounds with ESR2 are shown in Table 5. Finally, the following components have strong affinity with ESR2: anhydroicaritin (50.9932), 8-isopentenyl-kaempferol (50.8610), davalliosideA_qt (50.4315), xanthogalenol (49.4134), and linoleyl acetate (49.0539) and the results are shown in Figure 8.

**TABLE 5:** ESR2 molecular docking results.

NO.	ID	-CDOCKER_INTERACTION_ENERGY	Moleculer name	TCM
1	original ligand	52.872	THC	—
2	MOL004373	50.993	Anhydroicaritin	Yinyanghuo
3	MOL003542	50.861	8-Isopentenyl-kaempferol	Yinyanghuo
4	MOL009078	50.432	davallioside A_qt	Gusuibu
5	MOL009091	49.413	xanthogalenol	Gusuibu
6	MOL001645	49.054	Linoleyl acetate	Yinyanghuo
7	MOL004367	47.226	olivil	Yinyanghuo
8	MOL004380	44.539	2,7-Dihydrohomoerysotrine	Yinyanghuo
9	MOL000098	43.411	quercetin	Roucongrong; Yinyanghuo
10	MOL001040	42.865	(2 R)-5,7-dihydroxy-2-(4-hydroxyphenyl)chroman-4-one	Gusuibu
11	MOL004396	42.624	CID 12468616	Yinyanghuo
12	MOL000422	42.237	kaempferol	Gusuibu; Yinyanghuo
13	MOL000569	41.823	digallate	Gusuibu
14	MOL003044	41.674	Chryseriol	Yinyanghuo
15	MOL005190	41.543	eriodictyol	Gusuibu
16	MOL002914	41.484	Eriodyctiol (flavanone)	Gusuibu
17	MOL009087	41.415	marioside_qt	Gusuibu
18	MOL000006	41.160	luteolin	Gusuibu; Yinyanghuo
19	MOL001978	40.544	Aureusidin	Gusuibu
20	MOL004382	40.506	Yinyanghuo A	Yinyanghuo
21	MOL005320	40.259	arachidonate	Roucongrong
22	MOL007563	39.614	Yangambin	Roucongrong
23	MOL005384	39.439	suchilactone	Roucongrong
24	MOL000492	38.231	(+)-catechin	Gusuibu
25	MOL004328	36.992	naringenin	Gusuibu
26	MOL001792	36.862	Liquiritigenin	Yinyanghuo
27	MOL004427	36.796	Icariside A7	Yinyanghuo
28	MOL000622	36.103	Magnograndiolide	Yinyanghuo
29	MOL004388	34.448	CID 12115137	Yinyanghuo
30	MOL004391	34.278	8-prenyl-flavone	Yinyanghuo
31	MOL004386	28.124	Yinyanghuo E	Yinyanghuo
32	MOL004384	26.056	Yinyanghuo C	Yinyanghuo
33	MOL008871	24.876	Marckine	Roucongrong
34	MOL001510	18.730	24-epicampesterol	Yinyanghuo
35	MOL009061	17.976	22-Stigmasten-3-one	Gusuibu
36	MOL009076	16.461	cyclolaudenol	Gusuibu
37	MOL009075	15.128	cycloartenone	Gusuibu
38	MOL009063	5.975	Cyclolaudenol acetate	Gusuibu
39	MOL000358	−1.136	beta-sitosterol	Gusuibu; Roucongrong
40	MOL000359	−9.681	sitosterol	Yinyanghuo
41	MOL001771	−17.121	poriferast-5-en-3beta-ol	Yinyanghuo
42	MOL000449	−21.647	Stigmasterol	Gusuibu
43	MOL004394	No refined poses	Anhydroicaritin-3-O-alpha-l-rhamnoside	Yinyanghuo
44	MOL004425	No refined poses	Icariin	Yinyanghuo

In summary, the results showed that xanthogalenol in Gusuibu, arachidonate in Roucongrong, and anhydroicaritin in Yinyanghuo have good affinity for estrogen receptors. The proposed mechanism of WSYR in treating infertility was summarized in Supplementary Table S6, including the constituent herbs, compounds and corresponding functions.

## Discussion

By integrating the results of dry and wet experiments, it was found that the WSYR treats infertility by regulating hormone levels and inflammatory responses. Estrogen, estrogen receptors and various enzymes are closely related to clinical polycystic ovary syndrome, endometriosis and other reproductive endocrine diseases (Tang et al., 2019). Estrogen receptor is a ligand-activated transcription factor, which has several functional domains such as DNA binding domain, protein binding domain and transcriptional regulatory domain (Kuiper et al., 1996). Estrogen functions through ESR1 and ESR2 in follicle formation and ovulation. During the growth of follicles, ESR1 is mainly expressed on the follicle theca to regulate the proliferative effect of estrogen; while ESR2 is mainly expressed in the granulosa cells of the growing follicles at various stages to promote cell differentiation and retard proliferation (Pelletier and El-Alfy, 2000).

Various drugs by targeting estrogen receptors have been developed. For example, Clomiphene citrate (CC) is a clinically used estrogen receptor antagonistic drug to treat female and male infertility for 50 years (Roth et al., 2013). CC is an artificially synthetic estrogen derivative that can bind with estrogen receptor. CC antagonizes the hypothalamus-pituitary ER, leading to inhibition of the negative feedback effect of estradiol in circulation and increasing the pulse frequency of hypothalamus gonadotropin-releasing hormone. This leads to an increase in luteinizing hormone and follicle-stimulating hormone generated by the pituitary. Eventually, follicle growth and sperm generation are promoted (Revelli et al., 2011; Scovell and Khera, 2018; Quaas and Legro, 2019).

In our study, crystal complexes of ESR1 and ESR2 and their antagonists were selected to construct a molecular docking model to screen potential estrogen receptor antagonists in WSYR. The results revealed several compounds with high affinity to ESR1 or ESR2 and some of them have already been reported as related to estrogen regulation. For example, xanthogalenol in Gusuibu showed high affinity for both ESR1 and ESR2. Xanthogalenol may play a role in bone protection by activating the estrogen signaling pathway and promoting the differentiation and mineralization of osteoblasts *in vitro* (Wang et al., 2011). The arachidonate in Roucongrong showed high affinity for ESR1. It has been reported that arachidonate has pervasive modulatory effects on estrogen receptors in central and peripheral tissues (Kato, 1989). Anhydroicaritin in Yinyanghuo, which is the aglycone of Yinyanghuo’s indicator component icariin, showed high affinity to ESR2. As reported, anhydroicaritin has selective estrogen receptor regulatory activity (Tong et al., 2011). In addition, compounds such as marckine, yinyanghuo A, linoleyl acetate, suchilactone, davallioside A_qt, and 8-isopentenyl-kaempferol were also predicted by molecular docking. They may have potential estrogen-regulating effects and need further study.

In addition, WSYR was found with an anti-inflammatory role by our study. IL-4 and IL-10 are multipotent anti-inflammatory cytokines that mainly inhibit the proinflammatory processes. IL-4 signaling plays a part in not only Th2 cell function but also the regulation of regulatory T cells, which is essential in successful pregnancies. IL-10 mainly exerts anti-inflammatory effects by inhibiting proinflammatory cytokines such as IL-1, IL-12 and TNF. During normal pregnancy, IL-4 and IL-10 perform multiple functions, promoting placental formation and regulating trophoblast invasion and differentiation. Thus, the precise regulation of IL-4 and IL-10 is important for reducing maternal inflammation during different stages of pregnancy (Chatterjee et al., 2014). In this study, it was found that WSYR can exert an anti-inflammatory effect by upregulating the expression of *IL-4* and *IL-10*. Among them, Gusuibu, Roucongrong and Yinyanhuo upregulate the expression of *IL-4*, while Yinyanghuo upregulates the expression of *IL-4* and *IL10*. The role of the inflammatory markers was illustrated in Supplementary Figure S1.

This study integrates the methodical nature of dry experiments and the authenticity of wet experiments, systematically analyses the mechanism of action of WSYR in the treatment of infertility, and provides theoretical support and evidence for the clinical application of WSYR. However, the interaction of active compounds in WSYR and therapeutic targets needs to be further validated by *in vitro* experiments. Besides, since infertility is a complex disease, animal experiments are necessary to verify the regulatory role of WSYR on hormonal regulation and inflammatory responses *in vivo*.

## Data Availability

The datasets presented in this study can be found in online repositories. The names of the repository/repositories and accession number(s) can be found below: NCBI GEO, GSE202626.
